# Global regulatory policies for animal biotechnology: overview, opportunities and challenges

**DOI:** 10.3389/fgeed.2024.1467080

**Published:** 2024-09-24

**Authors:** Diane Wray-Cahen, Eric Hallerman, Mark Tizard

**Affiliations:** ^1^ Office of the Chief Scientist, U.S. Department of Agriculture, Washington, DC, United States; ^2^ Virginia Polytechnic Institute and State University, Blacksburg, VA, United States; ^3^ Commonwealth Scientific and Industrial Research Organisation, Geelong, VIC, Australia

**Keywords:** gene transfer, genome editing, biotechnology policy, regulatory oversight, commercialization

## Abstract

Genome editing (GnEd) has the potential to provide many benefits to animal agriculture, offering a means for achieving rapid growth, disease resistance, and novel phenotypes. The technology has the potential to be useful for rapidly incorporating traits into existing selectively bred animals without the need for crossbreeding and backcrossing. Yet only four products from animals created via biotechnology, all growth-enhanced fishes, have reached commercialization and only on a limited scale. The past failure of genetically engineered (or GM) products to reach conventional producers can largely be attributed to the high cost of meeting GMO regulatory requirements. We review the history of GMO regulations internationally, noting the influence of Codex Alimentarius on the development of many existing regulatory frameworks. We highlight new regulatory approaches for GnEd organisms, first developed by Argentina, and the adoption of similar approaches by other countries. Such new regulatory approaches allow GnEd organisms that could have been developed by conventional means to be regulated under the same rules as conventional organisms and in the future is likely to enhance the opportunity for biotech animals to enter production. Treating certain GnEd products as conventional has had a large impact on the variety of biotechnological innovations successfully navigating regulatory processes. We suggest that for the full potential of GnEd technologies to be realized, enabling public policies are needed to facilitate use of GnEd as a breeding tool to incorporate new traits within existing animal breeding programs, rather than only a tool to create distinct new products.

## 1 Introduction

Today, farmers face unprecedented challenges globally. Animal agriculture needs to be better prepared to combat climate change and other emerging threats, while increasing agricultural production and reducing its environmental footprint. Animal agriculture researchers and breeders are committed to transforming the future of our food system, making it more resilient, while lowering the environmental impact. Traditional selective breeding has made great strides through incremental changes, but improvement can be slow, requiring many generations (often years, sometimes decades). Addressing the current challenges requires a step-change - i.e., a large, sudden improvement - and more rapid response. Scientific advances in genomics, assisted reproductive technologies, and genetic marker-assisted breeding are being utilized to improve the pace of genetic improvement, but more tools are needed. Against this background, genome editing (GnEd) could significantly contribute to providing that step-change and offering solutions to many of these challenges; indeed, in some cases, GnEd may provide the only viable solution. As described in other papers within this special issue and reviewed in depth by Van Eenennaam (2023), GnEd of agricultural animals shows great promise for achieving animal agriculture goals such as improved yield, environmental protection, adaptation to climate change, disease and pest control, improved animal welfare, enhanced food quality and safety, and better control of reproduction. GnEd provides many opportunities for animal agriculture that have not been possible in the past. While not a silver bullet, integration of GnEd tools into animal breeding programs has the potential to be revolutionary. Animal breeders have just begun to realize some of its potential. However, for this potential to be fully realized, current regulatory approaches will need to be amended to become globally compatible.

With the development of GnEd techniques, animal breeders have renewed hope that biotechnological tools will become available to incorporate new traits into agricultural terrestrial and aquatic species in a way that did not occur with transgenic recombinant DNA (rDNA) technologies. The evolving global regulatory landscape has provided encouragement. However, it remains important to recall the past, the previously hopeful times, the lost opportunities, and the regulatory landscapes for transgenic rDNA technologies. It is also important to note that some GnEd end products that are cisgenic rather than transgenic may fall under the scope of regulatory requirements for genetically modified organisms (GMOs). It is also important to recognize that GnEd is a technique that, like rDNA techniques, can be used to create transgenic animals and therefore a sharp line cannot be drawn between all GnEd organisms and those organisms created via rDNA technologies and categorized as GMOs. This review will focus on how different countries approach the regulation of animals created via biotechnologies. We also note that terms and definitions used for biotechnologies and with their associated regulations (Box 1) may vary between countries.

Box 1 **| Key definitions used in this review**

**Cisgenic**–Adjective, describing the condition of an animal bearing a gene from its own species through human intervention.

**CRISPR**–stands for “clustered interspaced short palindromic repeats”. A gene-editing technology involving a guide RNA matching a desired target gene and an associated protein, such as Cas9 (CRISPR-associated protein 9), an endonuclease which causes a double-stranded DNA break, allowing targeted modifications of the genome.

**Gene or genome editing (GnEd)** – A group of technologies that give scientists the ability to change an organism’s DNA, allowing genetic material to be added, removed, or altered at particular locations in the genome.

**Gene transfer**–the technique of introducing a gene under novel transcriptional control into a host. The gene may have originated from the same or another species.

**Genetically modified (GM)** – adjective, refers to an organism whose genotype has been modified by application of biotechnology (e.g., gene transfer or chromosome set manipulation).

**GMO**–genetically modified organism; narrowly defined, the term connotes an organism that has been subject to classical gene transfer or one of its descendants bearing the transgene.

**Knockout** - Inactivation of a gene by homologous recombination following transfection with a suitable DNA construct.

**Null segregan**t–An individual whose ancestor bore a transgene or a genomic edit, but who does not carry that heritable modification.

**Recombinant DNA (rDNA) animal**–An animal in which the genetic material has been changed through recombinant DNA techniques, including direct injection of nucleic acid into cells or organelles.

**SDN**–site-directed nuclease.

**SDN edits**–Different classes of genome edits distinguished by on the basis of the size and nature of the edit, in particular whether a new DNA sequence is inserted into the host genome. Distinctions between the respective types of edits are not necessarily clear-cut, especially the distinction between SDN-2 and -3.

**SDN-1 edit**–A gene edit that produces a double-stranded break in the host genome without addition of “foreign” DNA; host-mediated repair of this break can lead to a mutation or deletion, causing gene silencing, gene knock-out, or a change in the activity of a gene.

**SDN-2 edit**–A gene edit that produces a double-stranded break, and a small nucleotide template is supplied that is complementary to the area of the break, which is used by the cell to repair the break. The template contains one to several small sequence changes in the genomic code Maryansky, 2006, of which the DNA repair mechanism copies into the host genome, resulting in a mutation of the target gene. SDN-1 and SDN-2 mutations can be as specific as the editing of a single base.

**SDN-3 edit**–A gene edit that induces a double-stranded break in the DNA but is accompanied by a template containing a gene or other sequence of genetic material. The cell’s DNA repair system utilizes this template to repair the break, resulting in the introduction of new genetic material.

**Transgene**–A gene construct bearing a gene from another species that was introduced into that organism by human intervention.

**Transgenic**–Adjective, describing the condition of an animal bearing a gene from another species through human intervention.

## 2 Regulatory oversight of animal biotechnology - A historical perspective

Regulations serve multiple roles, first and foremost to protect public health and safety, with a key goal being to prevent products with potentially harmful components from reaching the market. Regulations also can help instill public trust, whether in the food supply or in new drugs, vaccines, or biomedical devices. In addition, effective regulatory approaches that are transparent, science-based, and risk-proportionate can encourage and foster innovation.

The development of regulatory processes that encourage innovation and foster the process of bringing new products to the market in the rDNA-based agricultural biotechnology arena were met with extremely limited success, especially applications for animal agriculture. Regulations and their implementation play a critical role in determining how new technologies can be applied, what type of products or services are made available, and who can afford to access and benefit from these technologies. Regulations adopted over the last 50 years (since the Asilomar Conference on Recombinant DNA in 1975, Berg, 2008) have shaped the use, or in many cases the lack of use, of biotechnologies in food production, particularly in animal agriculture.

One challenge for food and agricultural applications of biotechnology has been that relevant regulatory processes have not been proportionate to the risks posed. It seems likely that the discomfort of the public with the use of new biotechnologies for food, along with successful anti-GMO disinformation campaigns, intensified pressure on regulatory bodies to require a higher level of regulatory oversight for food and agricultural use of biotechnology in the context of risks posed by transgenic rDNA. This was noted by the past Biotechnology Coordinator for the U.S. Department of Health and Human Services (HHS) Food and Drug Administration (FDA) the during an interview in which he stated that:

“*The foods developed by this [rDNA] technology undergo far more testing than all the other foods that enter the grocery store, for food safety. There’s really a huge burden that’s placed on the developers to use this technology, and that is going to be an issue for developing countries and an issue for small companies. It is, in fact, scientifically difficult to justify a lot of the testing that is being done today for these foods in terms of the public health issues that they actually don’t raise. But most of this is now being done to provide confidence to the public that the foods are safe*.” (Maryanski, 2006).

While this quote refers to biotech plants, regulations applied to the approval processes for animal biotechnology have been even more challenging for developers than those for plants. Although GM plants have faced significant regulatory challenges, many plant products have reached the market and for some crops, the majority of the seeds planted are derived from rDNA technology, with 72 countries currently growing or importing GM crops (ISAAA, 2024).

### 2.1 GMO (rDNA) laws, regulations, and guidance documents

Countries began considering regulatory processes for research involving rDNA and for products of rDNA technologies after the 1975 Asilomar Conference on Recombinant DNA (Berg, 2008). The United States was a leader in the development of guidelines and regulatory processes for research. The U.S. guidelines for rDNA research (NIH, 1976) went into place the year after Asilomar, and 10 years later were updated to include both plants and animals (NIH, 1986).

Although the first genetically engineered animal (a mouse, Gordon et al., 1980) was created before the first genetically engineered plant (a tobacco plant, Bevan et al., 1983), the regulatory process for plants was established much sooner. Development of regulatory processes and guidelines for rDNA, GM, and/or transgenic technologies was initiated in many countries in the 1980s and 1990s, and additional countries continue to develop and/or amend their regulatory processes for food and agricultural applications of biotechnology. For plants, the first approvals for food products occurred in the mid-1990s, with the Flavr Savr tomato and the disease-resistant Rainbow papaya (a rare example of a publicly funded biotech solution for an agricultural threat that navigated the regulatory process). In the United States, the U.S. Department of Agriculture’s (USDA) Animal and Plant Health Inspection Service (APHIS) released a regulatory process for rDNA plants in 1992 and HHS-FDA released their guidelines for food from rDNA-derived plants in 1994. The first food from a plant created via rDNA technology (the Flavr Savr tomato) appeared on the U.S. market that same year, only 7 years after it was first created in the laboratory; approval in Canada (1995) and the European Union (1996) quickly followed. Between 1994 and 1999, HHS-FDA made 80 food-safety determinations for plants containing rDNA traits, most conferring herbicide tolerance or insect resistance (US FDA, 2024). (For timelines of scientific and regulatory developments, see Hallerman et al., 2022).

### 2.2 Harmonization of regulatory systems - The importance of Codex

Moving into the 2000s, there was a growing need for enhanced alignment amongst regulatory processes across countries. Given the importance of international trade in agricultural products, it is important that the respective national regulatory systems be compatible. The purpose of the Codex Alimentarius Commission is to protect the health of consumers and ensure fair practices in the food trade (FAO and WHO, 2008a). Created in 2003, the Codex Alimentarius “Principles for the Risk Analysis of Foods Derived from Modern Biotechnology” (FAO and WHO, 2011) is the key document guiding global regulatory harmonization. Although there are differences among regulatory frameworks, there is general agreement on what is needed for safety evaluations of products for which there is insufficient familiarity or knowledge or those products which may raise potential specific risk concerns. Codex Alimentarius has proven influential for the development of biotechnology regulatory authorities in many countries. The development and acceptance of Codex may be given credit for the adoption of similar requirements across countries for rDNA (GM) products, helping to standardize food safety assessments and therefore, facilitating trade in products of plants derived from rDNA biotechnology.

The Codex “Guideline for the Conduct of Food Safety Assessment of Foods Derived from Recombinant-DNA Plants” (CXG 45-2003) and “Guideline for the Conduct of Food Safety Assessment of Foods Produced Using Recombinant-DNA Microorganisms” (CXG 46-2003) were both released in 2003 (FAO and WHO, 2011) and the “Guideline for the Conduct of Food Safety Assessment of Foods Derived from Recombinant-DNA Animals” (CXG 68-2008) was established in 2008 (FAO and WHO, 2008b). The Guideline recommends an approach for food safety assessment where a conventional counterpart exists and identifies the key elements for food safety and nutritional assessments (see Box 2). It does not address animal welfare; ethical, moral, or socioeconomic aspects; or environmental risks. The Guideline also does not address the “efficacy” of the trait; however, the Guideline does address an assessment of the impact of any antibiotic marker genes on therapeutic efficacy of orally administered antibiotics.

Box 2 **| Codex Alimentarius–The Guideline for the Conduct of Food Safety Assessment of Foods Produced Using Recombinant-DNA Animals was published in 2008 (FAO and WHO 2008).**

A. General description of the recombinant-DNA animal.

B. Description of the recipient animal prior to the modification and its use as food or for food production.

C. Description of the donor organism or other source(s) of the introduced recombinant DNA.

D. Description of the genetic modification(s) including the construct(s) used to introduce the recombinant-DNA.

E. Description of the methods used to produce the initial recombinant-DNA animal and the processes to produce the recombinant-DNA animal.

F. Characterization of the genetic modification(s) in the recombinant-DNA animal.

G. Safety assessment:

   a) Health status of the recombinant-DNA animal.
   b) Expressed substances (non-nucleic acid substances),
   c) Compositional analyses of key components,
   d) Food storage and processing, and
   e) Intended nutritional modification;

H. Other considerations.

The initial establishment of regulatory processes for rDNA animals to enter the food supply took longer than for plants and it was not until 2009 that the first regulatory process for animals with rDNA constructs was released. As with plants, the United States was the first country to publish its regulatory process guidance for animals created via rDNA technology (US FDA, 2009) and the first to approve a GM animal for food, the AquAdvantage salmon (US FDA, 2015). Although the AquAdvantage salmon was created only 2 years after the Flavr Savr tomato, its approval for food use came more than two decades after the Flavr Savr tomato had entered the market in the United States.

## 3 National regulatory policies

While most countries have GMO laws, there is likely no single “best” approach. In the global landscape, variation in regulatory approach, law making and legal enabling authority, and regulatory and political philosophies prevent identical regulation of GMOs. For example, regulatory triggers can be product-based (e.g., Canada) or process-based (as with GMO laws; e.g., Argentina) or use unique triggers (e.g., the United States). In addition, oversight of GMOs is different across countries, varying by roles of different authorities and/or ministries, and some may have shared oversight by multiple ministries or even multiple countries, as with Australia and New Zealand for food safety. Examples of regulatory policies are described below.

### 3.1 United States: Use of existing laws

Unlike most other countries, the United States chose not to create new GMO laws for regulatory oversight of the products of biotechnology. In 1986, the U.S. government established the Coordinated Framework for the Regulation of Biotechnology, which was updated in 1992 and again in 2017 (USDA and EPA, 2017). For food and agricultural products, the primary regulatory agencies involved are APHIS and Food Safety Inspection Service within USDA, HHS-FDA, and the Environmental Protection Agency (US-EPA). Under the Coordinated Framework, these individual U.S. regulatory agencies issue regulations and guidance documents to implement their individual pre-existing laws. HHS-FDA regulates heritable “intentional genomic alterations” (IGAs) in animals under the animal drug provisions of the U.S. Food, Drug, and Cosmetics Act.

The U.S. regulatory process is the same for all regulated uses of these animals (including use as food, production of biopharmaceuticals, and production of products for xenotransplantation). The regulated article is an IGA in an animal; this meets the FDA broad definition of a drug, which is a (non-food) article “that is intended to alter the structure or function” of the animal and/or is “intended to diagnose, cure, mitigate, treat or prevent disease”. The recently updated HHS-FDA Guidance for Industry (GFI) #187B (US FDA, 2024b) describes the pre-market application requirements to include: product characterization, food safety, and environmental impact. In addition, there are also requirements relative to durability, and effectiveness, along with post-approval monitoring and “adverse event” reporting.

Certain insects are under USDA-APHIS’s plant-pest authority, including silkworms, which due to their low risk profile, may not require additional regulatory oversight. Traits for population control in mosquitos are under US-EPA jurisdiction.

The United States was the first country to approve an rDNA construct in an animal for food use (although Canada was the first to allow its marketing). The rDNA construct in the AquAdvantage Salmon was approved by the HHS-FDA in 2015, but a congressionally imposed ban blocking the import of fertilized eggs from Canada (or other products of genetically engineered salmon, such as salmon filets from Panama or Canada) was in place until 2019. In 2020, HHS-FDA approved a second rDNA construct in an animal for food, the GalSafe pig. This animal was developed for human xenotransplantation in the early 2000s and a food use application was added to the biomedical use regulatory approval package (Table 1).

**TABLE 1:** Global regulatory decisions for genetically modified (via rDNA) and genome-edited (GnEd) animals for food or agricultural use. The table includes both approvals and decisions not to require GMO approvals.

Country	Genetically modified (rDNA)	Genome-edited
Argentina	Various species and traits in Phase 1, but none deregulated or commercialized	Tilapia (myostatin knockout, 2018) Beef cattle (SLICK, 2020) Dairy cattle (polled, SLICK, 2020) Cattle (myostatin knockout, 2021) Undisclosed, various species
Brazil	2 Mosquito lines (population control, 2014, 2020) Fall armyworm (2021) Atlantic salmon (somatotropin 2021)	Tilapia (myostatin knockout, 2019) Cattle (myostatin knockout, 2021) Beef Cattle (SLICK, 2021) Dairy Cattle (SLICK, 2023) PRRS virus-resistant pigs (2024)
Canada	Pig (phytase, 2010 - environment decision only) Atlantic salmon (somatotropin, 2013 - environment, 2016 - food)	
Colombia		PRRS virus-resistant pigs (2023)
Japan	10 Silkworms (various traits, color, dye-retention)	Red sea bream (MSTN KO, 2021; 2022 - variants) Tiger pufferfish (leptin receptor knockout, 2021; variants, 2022) Olive flounder (leptin receptor knockout, 2023)
United States	Atlantic salmon (somatotropin, 2015) Pig (alpha-gal knockout, 2020) Silkworms: not a risk concern (USDA-APHIS)	Beef cattle (SLICK, 2022)
Viet Nam	Silkworms (containing spider silk gene)	

### 3.2 Canada: Novelty

Canada also chose not to enact new GMO laws and instituted a system in which the regulatory trigger was the novelty of the product. In 1993, Canada established its regulatory framework for biotechnology with the goal of using existing legislation and regulatory bodies to regulate products of rDNA technology. Depending on the application or use, multiple agencies may be involved in the process, including the Canadian Food Inspection Agency (CFIA), Health Canada, and Environment and Climate Change Canada (ECCC) (Health Canada 2024b).

Canada is the only country that has a totally product-based regulatory approach. Canada requires a pre-market safety assessment for agricultural biotechnology products, including animals, only if they are novel (i.e., express a new characteristic or modify an existing characteristic) and could therefore pose a new risk. Canada is unique in that “novelty” also covers conventional breeding, not just biotechnology.

Canada published its Guidelines for the Safety Assessment of Novel Foods in 1994. Canada defines “novel food” as a substance that does not yet have a history of safe use as a food. In the context of animal biotechnology, it is a food that comes from an animal that has been genetically modified so that the animal either: (1) shows characteristics that it didn’t before, or (2) doesn’t show characteristics that it did before, or (3) has one or more characteristic that no longer falls within the expected range (Health Canada, 2006). Health Canada publishes all completed safety assessments of novel foods (see Health Canada 2024a) and may also determine that a process or product type is no longer novel, as they did for High Pressure Processing (HPP) (Health Canada, 2013), where for the 10 years prior to this “non-novel” determination, plant and animal foods undergoing HPP to kill microbes required a Novel Foods safety assessment.

The Canadian Environmental Protection Act of 1999 was written with GM animals in mind, with regulatory guidance for animate biotechnologies, including livestock, fishes, and insects. Environment Canada was the first regulatory agency in the world to issue decisions on animals for agricultural production, first the EnviroPig in 2010, a pig that produced phytase in its saliva, and then the AquAdvantage salmon in 2013 (Table 1). The safety of foods from GM animals is assessed by Health Canada and the Canadian Food Inspection Agency under Novel Foods Regulations. Canada was the second country to approve the AquAdvantage salmon for food use (Health Canada, 2016), and then became the first country to have food on the market from an animal containing an rDNA construct.

### 3.3 Enactment of new GMO laws

Most countries around the world have chosen to enact new laws for regulatory oversight of GMOs, rather than utilize existing laws. Many countries also have created new legal enabling authorities for regulatory oversight of the products of biotechnology. Many countries began putting these GMO frameworks in place during the 1990s. GMO laws generally apply to all organisms (i.e., plants, animals, and microorganisms), although there may be different regulations put in place for oversight over different types of organisms or different uses. Not all countries that enacted GMO laws have drafted regulations that apply to animals; some have put in place regulations only for crops, although the laws may cover all organisms.

#### 3.3.1 Argentina

In 1991, Argentina put in place a regulatory framework for GMOs. There is oversight by multiple agencies with different roles. The Biotechnology Directorate was set up within the Ministry of Agriculture, Livestock and Fisheries to be responsible for overall coordination, and a new Advisory Technical Committee on the use of GMOs was established within the National Service of Agricultural and Food Health and Quality (SENASA) for food and feed safety. For environmental safety, the National Advisory Committee on Agricultural Biotechnology (CONABIA) was created within the Ministry of Agriculture, Livestock and Fisheries. In addition, the National Directorate of Agricultural Food Markets within the Ministry of Agriculture, Livestock and Fisheries has oversight over the commercialization of products. Although Argentina instituted regulations for biotech plants in the 1990s, regulations for animals for food and agricultural use took longer. In 2017, Resolution 79-E/2017 updated Argentina’s regulations so that they were applicable for risk assessments for GMO animals. Argentina has not yet approved any GM animals for commercialization for food use.

#### 3.3.2 Brazil

In 1995, Brazil put in place its First Biosafety Law and GMO regulations. In 2005, Brazil passed a New Law for governing GMOs, creating a new National Biosafety Council (CNBS) and establishing the National Biosafety Policy for GMOs. They also restructured the National Biosafety Technical Commission (CTNBio) within the Ministry of Science, Technology and Innovations to be responsible for regulation of biotechnology. Multiple agencies may be involved in the approval and commercialization process, including the Ministry of Science, Livestock and Food Supply (MAPA); the National Agency for Sanitary Surveillance (ANVISA) in the Ministry of Health; the Brazilian Institute of Environment and Natural Resources (IBAMA) in the Ministry of Environment; and the Ministry of Fishing and Aquaculture (MPA). GM animals are regulated under the same legislation as GM plants, as are animal vaccines created via biotechnology.

In 2009, Brazil passed Normative Resolution No. 7 for the environmental release of GM animals, and in 2014, CTNBio issued its first approval for environmental release of a GM mosquito. In 2021, Brazil became the third country to approve the AquAdvantage salmon, although as of this date, it has not yet been commercialized within the country.

#### 3.3.3 Australia and New Zealand - Shared Responsibility for products

Australia and New Zealand have each enacted GMO laws (2023L). Given the close relationship between the two countries, they have chosen to implement a system in which they have shared responsibility for food safety and independent responsibility for approval of environmental release and impact assessments.

Food Standards Australia New Zealand (FSANZ) develops food standards for Australia and New Zealand. The FSANZ Code is enforced by state and territory departments, agencies, and local councils in Australia. The authority for imported food is with the New Zealand Ministry for Primary Industries and the Australian Department of Agriculture and Water Resources.

Environmental assessments are conducted separately, and each country has different laws and regulations governing them. In Australia, this is under the jurisdiction of the Office of the Gene Technology Regulator (OGTR) and in New Zealand, it is with the Environmental Protection Authority (NZ-EPA).

#### 3.3.4 Other countries with GMO laws

Japan applies the same GMO regulations that they use for GM plants to GM animals. Japan’s Ministry of Agriculture, Forestry, and Fisheries (MAFF) applies its “Law Concerning the Conservation and Sustainable Use of Biological Diversity through Regulations on the Use of Living Modified Organisms” and under the Food Sanitation Act, the Ministry of Health, Labour and Welfare (MHLW) is responsible for the food safety aspect of GM animals. Japan has approved a variety of GM silkworms with different traits, including various fluorescent colors and improved silk staining qualities (Table 1).

The European Union (EU) also has GMO regulations. The EU regulatory framework for GMOs applies to animals, as well as plants and microbes. The biotechnology approval process within the EU separates the risk assessment and risk management phases. The European Food Safety Authority (EFSA) conducts the scientific risk assessment phase and the risk management phase involves the European Commission’s Directorate General for Health and Food Safety (DG SANTE), the Council of the EU, and the European Parliament, hence the latter phase is more politically influenced. EFSA has created guidance documents on risk assessment for food and feed, and for animal health and welfare (EFSA Panels on Genetically Modified Organisms and Animal Health and Welfare, 2012) and on environmental risk assessment (EFSA Panel on Genetically Modified Organisms, 2013) for genetically modified animals. However, they have received no applications through their process for GM animals, which includes insects.

Other countries, such as the Philippines and Kenya, are in the process of creating regulations that cover GM animals.

### 3.4 Regulatory oversight of biotech animals in context

GM animals–including insects–that have received approval for food and agricultural applications in different countries are listed in Table 1; all of these were created using rDNA technology, although it should be noted that GnEd can be used to create transgenic/GM animals. In addition to biotech insect decisions in the United States, Brazil and Japan, Vietnam has made regulatory decisions that have allowed the commercialization of silkworms with rDNA constructs whose expression alters the characteristics of the silk.

To date, only one GM food animal has been commercially produced and marketed–the AquAdvantage Atlantic salmon. The GalSafe pig, while approved, is not being sold, but rather the company has donated meat from these animals to individuals suffering from Alpha-Gal Syndrome, a red meat allergy. None of the approved GM animals can be produced on conventional farms or aquaculture facilities. These GM animals must be produced in facilities approved by regulatory authorities and cannot be raised in general agricultural production.

There is a striking contrast between the approval and use of biotech crops and biotech animals. The reasons are complex. It took much longer for countries to begin putting in place regulatory processes for biotech animals. In addition, when compared with biotech crops (and biotech cell-based meat), regulatory oversight and requirements differ, the timeline to approval is much longer, and the associated costs, from research and development to approval, are much greater for biotech animals. As a result, for many decades it has been the case that fewer agricultural research funding resources are devoted to animal research than to crop research, whether for study of conventional or biotechnological approaches, disincentivizing to a great extent public and private investment which are needed to move science and adoption forward.

For example, in the United States, the path to commercialization for GM crops was defined in 1992. The first USDA-APHIS approvals for biotech crops occurred in the same year, followed by HHS-FDA decisions for biotech crops only 2 years later. In contrast, a path to commercialization for GM animals in the United States was not defined until 2009, 17 years after GM crops and importantly, after multiple funding organizations stopped supporting animal biotech research. Unlike for biotech crops, the first regulatory approval for a GM animal was more than 6 years after the regulatory guidance documents were published, which was followed by the aforementioned government-imposed import ban that prevented commercialization of the product within the United States until 2019. In contrast, 177 million acres of biotech crops were planted around the world in 2019. Therefore, despite active biotech research programs successfully producing needed traits in animals via rDNA technologies in the late 1990s and early 2000s, only one GM fish and one biomedical GM pig have been approved for food use in the world, and neither has been approved for use on conventional farms.

#### 3.4.1 Additional challenges faced by food animal applications of biotechnology

Crop farmers around the world have benefited from rDNA technology in GM plants, both in developed countries and also in many developing countries. Approximately 17 million farmers in 29 countries grow transgenic biotech crops, and 72 countries grow or import transgenic crops (ISAAA, 2019). The same is not true for animal farmers. The AquAdvantage Atlantic salmon remains the only animal in the world developed for food containing an rDNA construct to be approved and enter the marketplace. This GM fish has now been approved in just three countries, the United States, Canada, and Brazil (Table1). No other countries have made decisions for food animals containing rDNA constructs. The AquAdvantage Atlantic salmon can be legally grown only in facilities designed and approved specifically for this fish. Therefore, no conventional livestock, poultry, or fish farmers have been able to benefit from rDNA technology, beyond the use of the technology to produce new vaccines for the animals they raise.

Products developed for use in animal agriculture have also faced greater challenges than similar products for human biomedical use. This is perhaps best illustrated by rDNA hormone approvals. The first rDNA product approved for human use was insulin, created by Genentech in 1978, received HHS-FDA approval in 1982, and was on the market by 1983 (Genentech, 2016). Genentech created the first rDNA human growth hormone in 1981, and it was approved for human use in 1985. The first biotech product for animal agriculture to undergo a regulatory approval process was recombinant bovine growth hormone (somatotropin, rbST), also created by Genentech in the same year (1981) as human growth hormone. Monsanto applied for the approval of rbST; however, the rbST path to approval for animal use was much longer. Political pressure and vocal opposition to approval created a difficult environment for officials to conduct the regulatory process. HHS-FDA granted approval in 1993 amid anti-rbST protests and misinformation campaigns (Collier, 2000). At that time, some public health officials did not appear to understand the value of applying biotechnology for animal agriculture applications (Young, 2000).

Several factors in addition to technical and regulatory issues–including the structure of livestock industries, lack of public research funding and investment, and concern about public acceptance–have impeded commercialization of biotech animals (Van Eenennaam et al., 2021). While the AquAdvantage Atlantic salmon is the only animal developed for food production using rDNA technology to reach the market, others were developed, but never progressed through approval to commercial production. Some examples using rDNA technology include: the EnviroPig, which had public-sector developers (Forsberg et al., 2003), and received an approval from Environment Canada, but never made it through the food approval process to market. Dairy cows created by USDA researchers to produce lysostaphin in their milk at levels capable of enhancing resistance to *Staphylococccus aureus* infection, a major cause of mastitis (Wall et al., 2005). Public-sector-created transgenic sows producing milk with demonstrated clear health and welfare benefits for piglets (Wheeler et al., 2001). For biotech animals, the regulatory path was unclear, the cost of meeting proposed regulatory requirements was too high, and the potential to recover those expenses was too low. The long and expensive path to approval for the AquAdvantage salmon discouraged commercial breeding companies and venture capital companies from investing in animal biotech applications with a food and agriculture focus. In addition, there were concerns about public perception, despite the clear potential of some traits for improving animal health and welfare and reducing environmental impacts. For example, the Enviropig was the focus of anti-GMO campaigns (Sanderson, 2015).

For plant agriculture, the global regulatory environment helped to drive the decisions made by plant breeders and developers to focus only on two high-return traits (insect resistance and herbicide tolerance) for a few high-value row crops that could guarantee a return on the considerable investment required to meet the requirements of the regulatory process. This regulatory environment also created a situation where only a few large multinational crop breeding companies could afford to navigate these regulatory processes and bring products to market. For animal agriculture, none of the large breeding companies chose to invest in rDNA-created traits. This is still the case for newer GnEd technologies, for animal traits that may have to undergo the full biotech approval process (e.g., those that are transgenic). Few animal breeding companies or organizations have the means to assume the risk and cost of the GMO approval process and thus far only one large multinational animal breeding company has announced that it is pursuing full “GMO” approval for a new disease-resistance animal trait in swine. As with some of the rDNA traits of the 1990s and 2000s mentioned above, valuable agricultural traits developed via GnEd that are regulated as “GMOs” in key production and/or importing countries are likely to remain lost opportunities. Economic modeling of the costs associated with delayed commercialization of biotech livestock suggested billions of dollars in opportunity costs and reduced global food security (Van Eenennaam et al. (2021).

## 4 Changing scientific and regulatory landscapes

While creation of animals containing rDNA constructs has faced some technical challenges, advances in genome sequencing and the advent of GnEd technologies that allow targeted alterations to an existing genome have given rise to a very different scientific landscape than existed when the first transgenic food animal was created in 1985 and even after advances brought about by livestock cloning in 1996 and later refinements. Developments in GnEd technologies, especially the discovery of the CRISPR system and its relative precision and ease of use, have led to a scientific and technical renaissance. Regulators in many countries have also recognized that while GnEd could be used to create transgenic organisms, most of the organisms being created using GnEd techniques were cisgenic or contained random mutations similar to those seen with conventional breeding processes. This, in turn, has led to a rethinking of regulatory approaches to biotechnology. The global regulatory landscape for biotechnologies has changed dramatically in recent years for both plants and animals. Table 2 presents an overview of the current situation for regulatory approaches for food and agricultural applications of GnEd animals around the world. The authors recognize the challenge of including such a table, as it can quickly become out of date as the situation is quite dynamic as new countries join the list each year. One source of updates is the USDA Foreign Agricultural Service GAIN Reports (https://gain.fas.usda.gov/#/home). Many Foreign Agricultural Service posts publish an annual report on agricultural biotechnology, and major updates to regulatory policies are reported in the GAIN system when countries put them in place.

**TABLE 2:** Global approaches to regulation of agricultural applications of precision biotechnology (genome editing).

Category defining questions	Categories of products of precision breeding techniques				Null segregant	
Could be obtained via “conventional cross-breeding” or via mutagenesis?	yes	yes	yes	no	yes	
Nucleic acid template?	no	short	long	yes	NA	
“Foreign” DNA (Synthetic or Transgenic)	no	no	no	yes	NA	

Country	Policy^c^	Distinctions among categories may vary with country				Null segregant	Decisions for GnEd animals
Central and South America							
Argentina^1^	Resolution 21/2021	Not GMO	Not GMO	likely Not GMO^a^	GMO	Not GMO	yes
Brazil^2^	Normative Resolution 16	Not GMO	likely Not GMO	likely Not GMO	GMO	Not GMO	yes
Colombia^3^	Resolution No. 22991	Not GMO	Not GMO	likely Not GMO	GMO	Not GMO	yes
Uruguay^4^	Decree No. 84/024	Not GMO	Not GMO	likely Not GMO	GMO	Not GMO	
North America							
Canada^5^	Novel Foods Laws (Product based)	Method of genetic modification does not determine whether safety assessment is required; ‘novelty’ of product is regulatory trigger for pre-market assessment					
United States^6^ (HHS-FDA-CVM)	United States does not have “GMO” laws; the U.S. Coordinated Framework for Biotechnology Products is based upon existing U.S. laws “Intentional Genomic Alterations” (IGAs) introduced into animals via biotechnology are regulated under animal drug laws						
	GFI #187 A/B (final/draft)	IGAs individually evaluated. FDA may grant “enforcement discretion” (no further review) or IGAs may go through a pre-market approval process; determinations may vary within SDN categories				Not “GMO”	yes
Asia							
Indonesia	Draft proposal	Not GMO	Not GMO	likely Not GMO	GMO	Not GMO	
Japan^7^ ^,^ ^8^	MAFF- Environment	Not GMO	GMO	GMO	GMO	Not GMO	yes
	MAFF- Animal Products	Not GMO	Likely Not GMO	Likely GMO	GMO	Not GMO	
	Ministry of Health, Labor and Welfare	Not GMO	Likely not GMO	Likely GMO	GMO	Not GMO	
Oceania							
Australia^9^ (OGTR)	environmental release	Not GMO	GMO	GMO	GMO	Not GMO	
Australia/NZ^10^ (FSANZ - food)	Proposal P1055	Not GMO	Likely Not GMO	Likely Not GMO	GMO	Not GMO	
New Zealand (EPA)	Current legislation -environmental release (Hazardous Substances and New Organisms Act, 1996)	GMO	GMO	GMO	GMO	Not GMO	
	new legislation proposed	Not GMO	Risk-tiering system proposed		GMO	Not GMO	
Africa and Middle East							
Ghana^11^		Not GMO	Not GMO	Not GMO	GMO	Not GMO	
Kenya^12^		Not GMO	Not GMO	Not GMO	GMO	Not GMO	
Malawi		Not GMO	Not GMO	Not GMO	GMO	Not GMO	
Nigeria^13^		Not GMO	Not GMO	Not GMO	GMO	Not GMO	
South Africa^14^	GMO Act of 1997	GMO	GMO	GMO	GMO	undetermined	
Europe							
Norway^15^	Proposed	Not GMO	expedited assessment	expedited assessment	GMO		
United Kingdom^16^ (DEFRA)	Implementation for animals after welfare provisions established	Not GMO	Not GMO	Not GMO	GMO	Not GMO	
Plant-specific							
Chile^17^		Not GMO	Not GMO	likely Not GMO	GMO	Not GMO	
China		4-tiered approach (classification criteria not yet clarified)					
Costa Rica^18^ ^,^ ^b^	Decree No. 44244-MAG	Not GMO	Not GMO	likely Not GMO	GMO	Not GMO	
Ecuador^19^ ^,^ ^b^	Ministerial Agreement No. 063	Not GMO	Not GMO	likely Not GMO	GMO	Not GMO	
European Union^20^	European Commission Proposal	Not GMO	Not GMO	Not GMO	GMO	Not GMO	
Guatemala^21^ ^,^ ^b^	Ministerial Agreement No. 271-2019	Not GMO	Not GMO	likely Not GMO	GMO	Not GMO	
Honduras^22^ ^,^ ^b^	Agreement C.D. SENASA 008-2019	Not GMO	Not GMO	likely Not GMO	GMO	Not GMO	
India^23^		Not GMO	Not GMO	Not GMO	GMO	Not GMO	
		SDN1	SDN2	SDN2	SDN3		
Israel		Not GMO	Not GMO	Not GMO	GMO	Not GMO	
Paraguay^24^ ^,^ ^b^	Resolution MAG No. 842/2019	Not GMO	Not GMO	likely Not GMO	GMO	Not GMO	
Philippines^25^	animal regulations under consideration	Not GMO	Not GMO	Not GMO	GMO	Not GMO	
Singapore^26^	Singapore Food Agency (SFA) and GMAC	Not GMO (notify SFA)	Not GMO (notify SFA)	likely Not GMO (notify SFA)	GMO	Not GMO	
Thailand^27^ ^,^ ^b^	Ministry of Agriculture and Cooperatives B.E. 2567	Not GMO	Not GMO	likely Not GMO	GMO	Not GMO	

^a^ perfect allelic replacement.

^b^ no GM, animal regulations in place; based on the definition of an “LMO”.

SDN: Site-Directed Nuclease.

GMO: genetically modified organism; “GMO” and “Not GMO” indicate whether an organism in each category would be included in a country’s rDNA/GMO, regulations.

(NB: not all countries use the term “GMO” or have GMO, laws. Quotes are used to indicate these instances and whether products would be subject to biotechnology regulations).

^c^ **FAS GAIN:** https://gain.fas.usda.gov/#/(updated annually) Details on country policies available in the USDA GAIN system and in the weblinks below.

**Weblinks to Country Policies.**

1 Argentina: https://www.argentina.gob.ar/agricultura/alimentos-y-bioeconomia/nuevas-tecnicas-de-mejoramiento-nbt; https://www.argentina.gob.ar/normativa/nacional/resoluci%C3%B3n-21-2021-346839

2 Brazil: https://ctnbio.mctic.gov.br/en/resolucoes-normativas/-/asset_publisher/OgW431Rs9dQ6/content/resolucao-normativa-n%C2%BA-16-de-15-de-janeiro-de-2018

3 Colombia: https://www.anla.gov.co/eureka/normatividad/resoluciones/2960-resolucion-no-22991-del-11-de-noviembre-de-2022

4 Uruguay: https://www.impo.com.uy/bases/decretos/84-2024

5 Canada: https://www.canada.ca/en/health-canada/services/food-nutrition/genetically-modified-foods-other-novel-foods.html; https://inspection.canada.ca/en/plant-varieties/plants-novel-traits/gene-editing-techniques

6 United States: https://www.fda.gov/animal-veterinary/biotechnology-products-cvm-animals-and-animal-food/intentional-genomic-alterations-igas-animals

7 Japan (MAFF): https://www.maff.go.jp/j/syouan/nouan/carta/tetuduki/nbt.html

8 Japan (MHLW): https://www.mhlw.go.jp/stf/seisakunitsuite/bunya/kenkou_iryou/shokuhin/bio/genomed/index_00012.html

9 Australia (OGTR): https://www.ogtr.gov.au/resources/publications/overview-status-organisms-modified-using-gene-editing-and-other-new-technologies; https://www.genetechnology.gov.au/reviews-and-consultations/past/2017-third-review

10 Australia/NZ (FSANZ): https://www.foodstandards.gov.au/food-standards-code/proposals/p1055-definitions-for-gene-technology-and-new-breeding-techniques

11 Ghana: https://bch.cbd.int/en/database/LAW/BCH-%20LAW-GH-265861-1

12 Kenya: https://www.biosafetykenya.go.ke/images/GENOME-EDITING-GUIDELINES-FINAL-VERSION-25th-Feb-2022-03.pdf

13 Nigeria: https://nbma.gov.ng/wp-content/uploads/2022/03/NATIONAL-GENE-EDITING-GUIDELINE.pdf

14 South Africa: https://old.dalrrd.gov.za/doc/Minister%20final%20decision%20on%20AGBIZ%20appeal.pdf

15 Norway: https://www.bioteknologiradet.no/filarkiv/2019/01/Proposal-for-relaxation-of-GMO-regulations-with-annexes.pdf.

16 United Kingdom: https://www.legislation.gov.uk/ukpga/2023/6/contents/enacted

17 Chile: https://www.sag.gob.cl/ambitos-de-accion/aplicabilidad-de-resolucion-ndeg-15232001-en-material-de-propagacion-desarrollado-por-nuevas-tecnicas-de-fitomejoramiento

18 Costa Rica: https://www.imprentanacional.go.cr/pub/2023/11/10/ALCA222_10_11_2023.pdf

19 Ecuador: https://www.fao.org/faolex/results/details/es/c/LEX-FAOC223895/

20 European Union: https://ec.europa.eu/info/law/better-regulation/have-your-say/initiatives/13119-Legislation-for-plants-produced-by-certain-new-genomic-techniques_en

21 Guatemala: https://visar.maga.gob.gt/visar/2019/20/AM271-2019.pdf

22 Honduras: https://senasa.gob.hn/web/wp-content/uploads/2022/02/ACUERDO-CD-SENASA-008-2019-GACETA-35047.pdf

23 India: https://dbtindia.gov.in/sites/default/files/SOPs%20on%20Genome%20Edited%20Plants.pdf

24 Paraguay: https://conbio.mag.gov.py/media/ckfinder/files/RES.N842%2010%20DE%20JULIO%20DE%202019%20NBT.pdf

25 Philippines: https://www.da.gov.ph/wp-content/uploads/2022/06/mc08_s2022_Revised.pdf

26 Singapore: https://www.sfa.gov.sg/docs/default-source/food-information/guidance-on-regulatory-framework-for-genome-edited-crops-for-use-as-food-and-feed-(2024-08).pdf; https://www.gmac.sg/guidelines/?tab=singapore-guidelines-on-the-release-of-agriculture-related-gmos#tab_section

27 Thailand: https://apps.fas.usda.gov/newgainapi/api/Report/DownloadReportByFileName?fileName=Regulation%20on%20Plants%20Developed%20Using%20Genome%20Editing%20Technology%20_Bangkok_Thailand_TH2024-0052.pdf

### 4.1 Modernizing regulatory approaches

With new regulatory approaches, the goals for regulatory oversight for the products of biotechnology remain the same as for all foods, with the top priority being to protect the safety of humans, animals, and the environment. New regulatory approaches being developed around the world for products of GnEd are increasingly focusing on the characteristics and potential hazards of the products of new technologies, rather than on the method used to create them. These are aided by a better understanding of the associated molecular biology and genomics. Regulatory officials have long understood the importance of enabling regulatory processes that foster innovation and discovery in the biomedical realm, and there is an increasing awareness of the importance of innovation in the food and agricultural sector. There is a recognized need to encourage creation of new EPA NZ, (2024), innovative, safe agricultural tools and products that address growing global challenges and threats, as well as a need to facilitate more rapid integration of animals developed using precision breeding tools into breeding programs and allowing for their use by farmers within current production systems and husbandry practices. Provision for the safe use of these tools by farmers and breeders is expected to improve global food security, while also helping to meet global environmental and sustainability goals.

**FIGURE 1:**
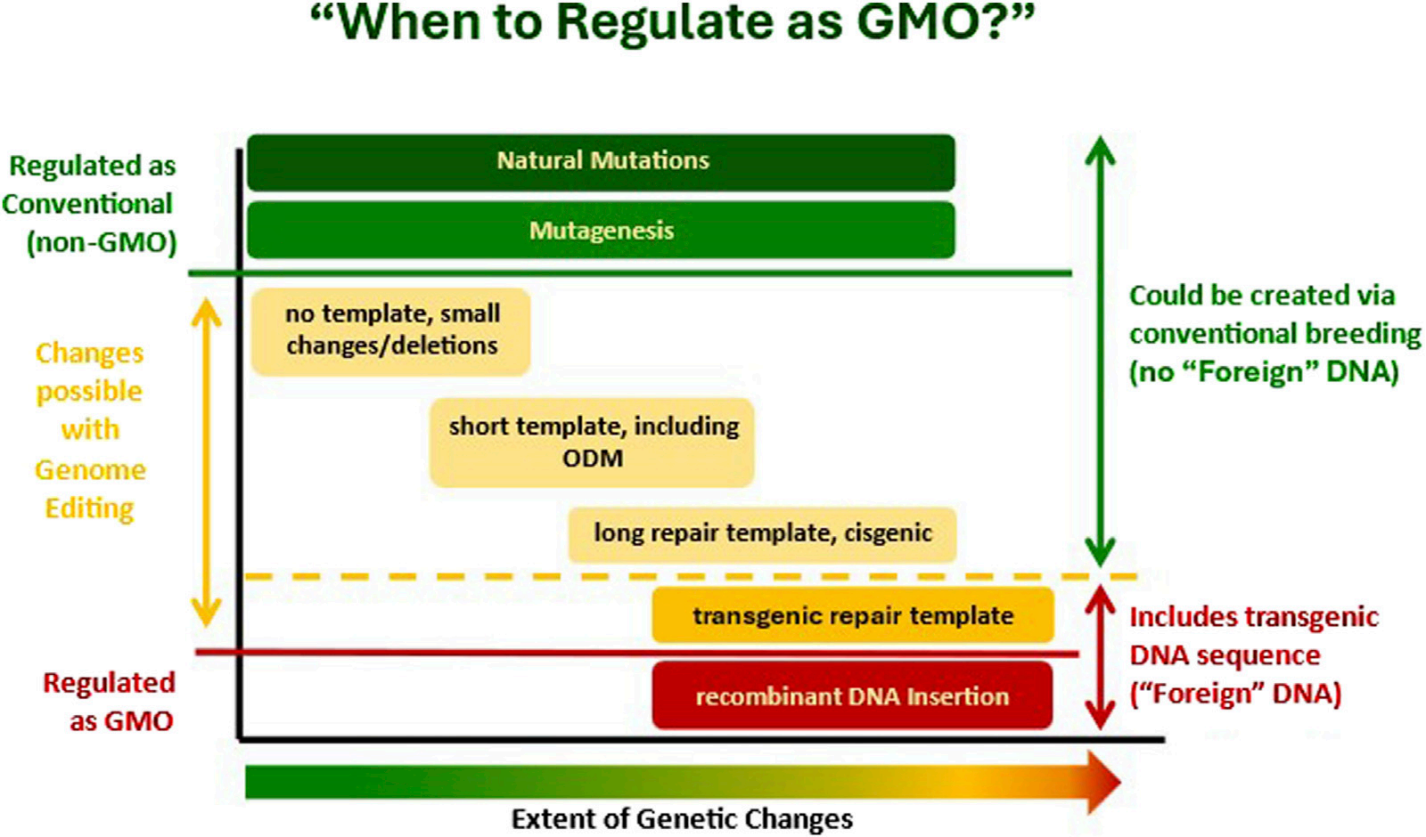
Conceptual view of consideration of when an organism should or should not be regulated as a GMO.

In the development of new regulatory approaches, the question that regulatory officials in many countries have asked is: When is additional regulation required for a product under their existing GMO laws? There is general agreement across countries that natural mutations and mutagenesis (shown in green on Figure 1) are not regulated as GMOs, but rather are regulated as conventional products (unless, in the case of Canada or perhaps the EU, if they are identified as novel). There is also agreement that for products where transgenes are inserted (shown in red on Figure 1), these products are regulated under GMO laws, with additional evaluations beyond those required for products from conventional organisms. The focus of the present dialogue is the categories in the middle (shown in yellow in Figure 1). The question has been one of where to draw the regulatory “GMO/non-GMO” line. For many countries, the line has been drawn where shown by the yellow dashed line in Figure 1, where below the line is templated repairs coding for “foreign” DNA, which would be regulated under GMO laws, and above the line are organisms that could have been created via conventional breeding, which would be regulated as conventional products.

### 4.2 Use of the definition of LMO in the Cartagena Protocol

In 2015, Argentina became the first country to publish this new approach to regulation of products created using new breeding technologies such as GnEd, an approach that excluded from GMO regulations products that did not incorporate “foreign” DNA (Whelan and Lema, 2015). The key question asked by Argentina was what constitutes a GMO within the context of their laws, specifically, what types of changes in DNA would result in the creation of a GMO. To address this question, they looked to the Cartagena Protocol on Biosafety (Convention on Biological Diversity, 2000) and its definition of “living modified organism” (LMO):

Article 3 (Use of Terms) of the Cartagena Protocol provides definitions for both LMO and “modern biotechnology”. Term (g) states that “living modified organism” means any living organism that possesses a novel combination of genetic material obtained through the use of modern biotechnology; and term (i) states that “modern biotechnology” means the application of: (a) *in vitro* nucleic acid techniques, including recombinant deoxyribonucleic acid (DNA) and direct injection of nucleic acid into cells or organelles, or (b) fusion of cells beyond the taxonomic family, that overcome natural physiological reproductive or recombination barriers and that are not techniques used in traditional breeding and selection.

Therefore, for an organism to be classified as an LMO, it not only requires the use of modern biotechnology, but also a “novel combination of genetic material”. Hence, Argentina and an increasing number of additional countries have created new regulatory policies for products of GnEd for which the critical point in the decision tree is whether the final product contains a new or novel combination of genetic material, sometimes worded as whether or not it contains “foreign” DNA (Figure 1). Organisms not defined as GMOs are regulated as conventional products and do not have the additional requirements for GMOs imposed upon them.

Following Argentina’s lead, an increasing number of countries with GMO laws have adopted the approach of applying the Cartagena Protocol’s definition of an LMO as the regulatory trigger, and new products that do not have “foreign” DNA incorporated into their genome are therefore regulated as conventional products. This approach is being adopted by many countries in Latin America, Africa, and elsewhere in the world (Table 2).

#### 4.2.1 Latin America

As noted above, **Argentina** has been a global leader in the development and implementation of regulations for the use of GnEd products. Their initial new breeding techniques (NBT) regulations were updated in 2020 and published as Resolution N°21/2021 in 2021. One innovative aspect of Argentina’s process is that it allows for regulatory consultation and pre-determinations when the product is at the design stage. The applicant must provide information on the methodology applied, genetic changes in the new product, new trait, and evidence of transgene deletion (if applicable), which must be verified after its development. The process is intended to allow a developer to better predict costs and time required for regulatory consideration of the product at the product-design stage. Argentina’s intention is to accelerate the speed of innovation for GnEd products relative to GMOs and allow developers to bring their product to market more quickly.

In 2018, Argentina made the world’s first determination that a GnEd animal that does not contain transgenic DNA is not a GMO (and that, as a deletion, could have been developed via conventional breeding) and therefore should be regulated as a conventional animal (Table 1). An increasing number of countries are beginning to put in place new regulatory approaches for products of GnEd, many similar to that of Argentina.

**Brazil**’s CTNBio issued Normative Resolution Number 16 in 2018, becoming the second country to publish a new regulatory approach for products generated by new breeding technologies, Brazil’s process also allows products, after case-by-case analysis, to be determined to be free of rDNA and/or DNA that is novel to the species and thus to be classified as non-GMO. To have a product classified as non-GMO, the developers would need to describe the technique(s) employed and the parental and product organisms. Developers also need to show that there are no non-negligible unintended effects (e.g., non-negligible off-target edits). Additionally, Normative Resolution Number 16 states a policy of precaution regarding gene drives.

Brazil made the world’s second determination that a GnEd animal that does not contain transgenic DNA is not a GMO (a myostatin knockout fish for which Argentina made the same determination). Brazil have reported more non-GMO decisions about GnEd animal products than any other country (Table 1). For commercialization within Brazil, the developers also need the approval of the specific regulatory agency, i.e., environmental, animal or human health agencies, relevant to commercial production of that product.

**Colombia** has a similar LMO definition-based regulatory approach for GnEd organisms as Argentina (Table 2). Like Argentina and Brazil, Colombia has made a non-GMO decision for GnEd animals (USDA-FAS, 2023e). This non-GMO decision by Colombia in 2023 was the first regulatory determination in the world for an animal with a disease-resistance trait, a PRRSv-resistant pig. In 2024, Brazil made a similar determination for the same GnEd animal (Table 1).

Other Latin American countries are adopting policies similar to Argentina (see Table 2).

#### 4.2.2 North America

In the **United States**, USDA-APHIS, HHS-FDA, and US-EPA have published new approaches for plants, exempting certain GnEd plant products. HHS-FDA’s Center for Veterinary Medicine (CVM) has also published a new guidance that applies to GnEd animals, GFI#187A (US FDA, 2024a). It describes a new approach where some food and agricultural applications of GnEd animals may be considered candidates for enforcement discretion (ED), which means that the developers would not need to submit an application for pre-market approval. Data expectations may vary depending on the genome edit (IGA, in HHS-FDA guidance) and its intended use, but it is expected that developers would submit information including the methodology used to generate the animal, characterization of the genomic sequence modified, and information addressing animal safety, food safety, and risk of impacts on the environment, as appropriate for the intended use. ED is considered on a case-by-case basis as opposed to categorically. HHS-FDA has made one decision to exercise ED for an IGA in a food use animal, the cisgenic genome edit of the prolactin receptor gene (SLICK gene) in two beef cattle, an edit that mimics a naturally occurring mutation that influences hair characteristics and subsequent tolerance to heat (US FDA, 2022; Table 1). The ED decision enables the developer to market the genetics from these animals without undergoing a pre-market approval process. ED is the same regulatory mechanism that HHS-FDA has used for GloFish–transgenic fluorescent aquarium fishes. In the United States, the genome edit in the PRRSv-resistant pig, which received a non-GMO decision in Colombia and Brazil (as it contains a deletion and does not have any transgenic sequences), is undergoing the new drug approval process for IGAs in animals (according to FDA’s GFI#187B).

As noted earlier, **Canada** has a novel foods approach, in which products do not require additional regulation unless they are novel. This is a product-based regulatory approach that applies to all products whether or not they are created with biotechnology. The Government of Canada (CFIA, 2020) has indicated that “Some products developed using gene editing techniques may not meet the regulatory definition of “novel”. If a product is not novel, it is considered equivalent to its existing counterparts, and no pre-market assessment is required.” Canada has not yet made any decisions for GnEd animals, however it has made determinations that certain GnEd crops are not novel.

#### 4.2.3 Asia and Oceania

**Japan** has a process where cisgenic GnEd organisms are generally regulated as non-GMOs and they have made non-GMO decisions for plants and animals (MHLW, 2021; Matsuo and Tachikawa, 2022), including for the first food from GnEd animals, as noted in Section 4.2.7 below.

**The Philippines** has a process in place for plants similar to Japan and Argentina and has made a non-GMO decision for a GnEd plant. The process for animals is currently under consideration.

**Australia** is currently in the process of reviewing their biotechnology codes for GnEd. In the meantime, they are exempting deletions from GMO regulations, whereas templated changes currently are regulated as GMOs.

In **New Zealand**, although regulators made an initial “non-GMO” ruling for some products of GnEd, courts struck this decision down, stating that as their current GMO regulations are written, GnEd products cannot be exempted from their GMO regulations. Now efforts are underway to bring New Zealand in closer alignment with other countries, such as Australia. In early 2024, the New Zealand Environmental Protection Authority clarified that null-segregants were not GMOs under the Hazardous Substances and New Organisms Act 1996 (EPA NZ 2024). In mid-2024, the New Zealand Government announced it will create a Gene Technology Regulator, guided by groups representing expert technical advisors, the Maori peoples and industry. It will be hosted by the Environmental Protection Authority, with an indication that specific (GnEd) gene technologies may be exempted from regulation (MBIE, 2024).

**Singapore** and **Thailand** have also recognized the advantages of GnEd and in 2024, both announced new policies. The Singapore Food Agency (2024) published a “Guidance on Regulatory Framework for Genome Edited Crops for Use as Food and/or Feed” . Like approaches by other countries, genome edits that do not contain “foreign DNA” are not required to undergo GMO pre-market approval. But developers are encouraged to notify the Singapore Food Agency, so that they may add it to a list of GnEd crops that are equivalent to conventional crops. Wider use in agriculture (i.e., livestock and aquaculture) may be considered. Thailand also released new guidelines for GnEd organisms, where those not containing a novel combination of genetic material or material from a non-sexually compatible host are determined to be non-GMO. Policies for plants have been developed and those for animals are expected to take longer.

#### 4.2.4 Europe

**Norway** has proposed a three-tiered approach with notification, expedited review, or standard review where “foreign” DNA insertions are regulated as GMOs. The Norwegian policy seems unlikely to move forward until the EU has finalized its own regulatory approach (NBAB, 2019).

However, like New Zealand, courts in the **European Union** (EU) have ruled that, as their current GMO regulations are written, GnEd products cannot be exempted from their GMO regulations. The EU has been exploring exclusions for some GnEd plants, and in early 2024, the European Parliament voted to exempt some types of new genomic techniques from GMO requirements; it is not yet clear what their approach will be for animals. A study on new genomic techniques (NGTs) published by the European Commission (EC, 2021) included the use of new genomic techniques in animals. However, because they had less information on animals than on plants, the Commission mandated that EFSA provide an opinion on new developments in biotechnology applied to animals, including synthetic biology and NGTs. This opinion is expected in 2025.

The **United Kingdom** also has moved forward with exclusions for GnEd plants, and processes for animals are under consideration.

#### 4.2.5 Africa

**Kenya**, **Nigeria**, **Malawi**, and **Ghana** have developed policies similar to those of Argentina, where only GnEd products with “foreign” DNA sequences in the final end product must go through the full GMO approval process. No animal decisions have yet been made, but there is on-going research with GnEd.

**South Africa** has moved in a different direction with their Minister of Agriculture, Land Reform, and Rural Development ruling that products of GnEd would be subject to regulations under their GMO Act of 1997.

#### 4.2.6 Other countries

Other countries are also developing draft policies, and a number of these are aligning with those of Argentina, Brazil, and Japan.

#### 4.2.7 Regulatory decisions for GnEd animals

Food and agricultural applications for GnEd animals for which regulatory decisions have been made in different countries are listed in Table 1. Currently the only food from GnEd animals on the market is in Japan. i.e., myostatin knockout red sea bream (Kishimoto et al., 2018) and leptin receptor knockout tiger puffer (Kishimoto et al., 2019), and olive flounder (Regional Fish Institute, 2023). The SLICK cattle have received decisions in a number of countries, confirming that the SLICK trait is cisgenic and that there is no need to require these animals to undergo a GMO approval process. However, products from GnEd cattle are not yet available to consumers.

## 5 Impact of two regulatory scenarios: Opportunities lost or gained

Regulations and how they are implemented and applied shape what products are developed and who can afford to bring the products of these new breeding technologies to the marketplace. There are two contrasting approaches to regulatory oversight–one where there are “no exclusions” and all products of biotechnology are regulated as GMOs and the second where there are “exclusions” for some biotech products where, for example, those that could have been created via conventional breeding are regulated as conventional products without the additional GMO regulatory requirements. These different regulatory approaches lead to different outcomes regarding the mix of biotechnology products that ultimately reach farmers.

Under the “no exclusions” approach, which is the *status quo* approach for some countries, the GMO rules apply to all biotech products. Under this approach, only large multinational companies, mostly engaged with marketing seed for major commodity crop plants, would be expected to be able to afford to navigate the regulatory process. Developers, then, would be expected to come from very few countries, and commercialized products would likely be dominated by row crops and high-return traits such as herbicide tolerance and insect resistance. Very few biotech food animals could be commercialized, and their production would be limited. Therefore, biotech animals could not be widely produced by farmers and their needs for quickly acquiring new traits would be unmet. In this scenario, many opportunities for commercialization of innovative animals would be lost.

In contrast, under the “exclusions” approach (where at least some GnEd animals are regarded as conventional animals), many new animal traits resulting from publicly funded research or from small and medium-sized enterprises (SMEs) could avoid additional GMO regulatory requirements. GnEd animals from more countries could be commercialized, and a relatively broad range of livestock, fruits, vegetables, and flowers, including products with consumer-oriented traits, could find their way into commercial production. Notable, quicker solutions to regional agricultural problems also could be implemented.

When GMO regulations were first established, it was with a “no exclusions” approach and as new “exclusions” approaches have been put in place, the impact of the change in regulatory procedure has become quite clear. In the United States, from 1992 through 2020, USDA-APHIS conducted “GMO” regulatory reviews of 136 petitions involving 19 crops; approximately three-quarters of these petitions were originated by large biotechnology companies, with relatively few by SMEs, and only five from publicly funded entities. Following the change of USDA-APHIS procedures to allow for the exclusion from biotech regulation of some GnEd products, the impact was dramatic (Wray-Cahen et al., 2022). From 2020 to this writing in 2024, of 67 regulatory status reviews involving 19 plants, over three-quarters originated SMEs, with equal smaller proportions from large companies and publicly-funded entities, in addition (as of 1 July 2024) there have been 95 Confirmation Letters for plants indicating that they are exempt from the regulatory status review requirements, over 90% have been from SMEs (https://www.aphis.usda.gov/biotechnology). After Argentina implemented their new NBT regulatory approach, they observed similar impacts on the type of submissions they received, including for animal applications (Whelan et al., 2020).

## 6 Challenges and opportunities for regulatory compatibility and cooperation

The products of animal agriculture are important in international trade, and it is important that trading partners have regulatory policies that foster the export and import of animal products. However, the achievement of regulatory compatibility and cooperation for export and import of the products of animal biotechnology faces significant challenges. Among them, GMO regulatory approaches for biotechnology for many countries were developed for crops or biomedical applications, and as noted above, development of approaches for livestock is incomplete. Further, there is the potential for misalignment of countries’ regulatory approaches for products of newer technologies.

Harmonization or compatibility of regulatory approaches among trade partners would facilitate export and import of the products of animal biotechnology. Most countries have similar GMO laws for all classes of organisms, covering both plants and animals, which can facilitate opportunities for harmonization. In this critical moment, more countries are considering what regulatory approach to apply to products of GnEd, and many are considering similar types of exclusions from GMO laws. Harmonization and alignment of regulatory approaches is promoted by regional and international cooperation, as is ongoing in the southern cone of South America, Central America, Africa, and Asia. Development of harmonized animal biotechnology regulatory policy has been discussed at the bilateral and multilateral levels. A community of researchers, developers and regulators has organized several international workshops aimed at elaboration and implementation of risk-proportionate, technology-enabling and compatible regulatory policies on animal biotechnology (https://www.isaaa.org/kc/proceedings/animalbiotechnology/default.asp).

## 7 A perspective on effective regulatory approaches

Against the background of the country-by-country review of agricultural biotechnology regulatory policy presented above, it becomes clear that this is a pivotal moment in the development of biotechnology regulatory policy, especially for food and agricultural products created using GnEd. Well-considered policies would enable safe products to reach the market, encourage development of new ideas and innovations that can be incorporated into standard breeding practices, and provide farmers with the choice of the best animal lines to meet current and future challenges more sustainably. To achieve these goals, regulatory approaches should be: (1) science-based, risk-proportionate, and defensible, (2) credible to the public, which may have non-scientific, values-based issues, (3) provide oversight that is timely and predictable, which is important for fostering innovation, (4) appropriate for intended use, e.g., food vs biomedical applications, and (5) transparent to all. Effective regulations should not only protect humans, animals, and the environment, they should also allow the production and marketing of safe products from animals created with the aid of agricultural biotechnology.

**FIGURE 2:**
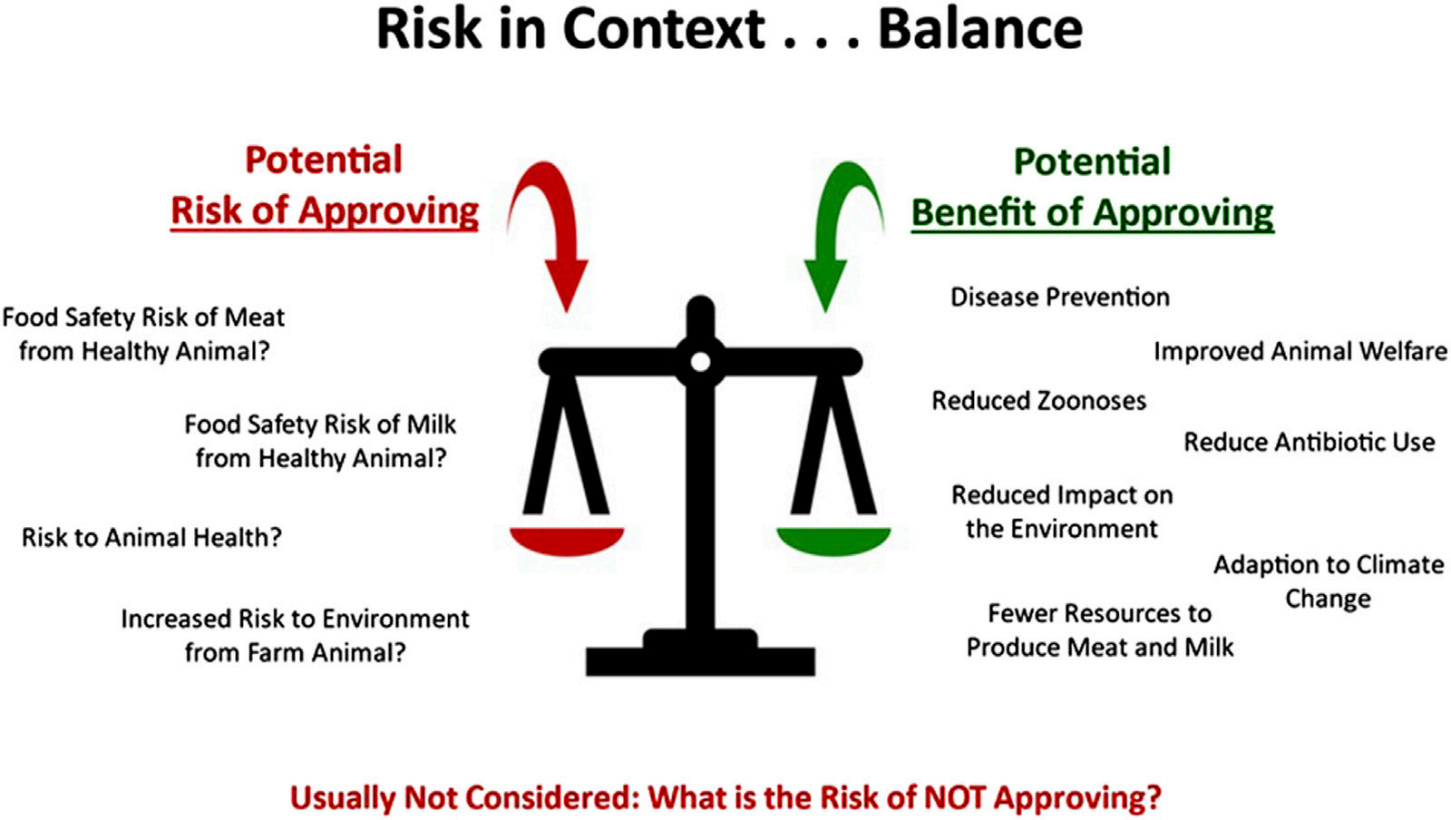
Potential risks of approving or not approving applications for biotech animals.

A critical balance must be struck between the risk associated with allowing biotech farm animals into production and the potential benefits that the biotech-introduced traits bring to animal production, as well as the potential loss of benefit suffered if new technologies are not able to be used in animal production (Figure 2). As we look to the future, we advocate for balanced risk-proportionate regulatory approaches.

### 7.1 Regulatory crossroads - Hope for the future

Faced with increasing pressure to produce animal products safely and sustainably, we seek to provide farmers with innovative solutions resulting not only from large corporations, but also from publicly-funded research groups and small- and medium-sized private-sector companies. As noted above and elsewhere in this volume, solutions from new animal breeding technologies such as GnEd are becoming available to address challenges posed by climate change and disease and pest threats, and that address animal welfare and food security issues. Unfortunately, the promise of agricultural biotechnology has not yet been well realized for the animal production sector. In the past, many innovative and potentially impactful research products using older biotechnologies were developed but failed to reach farmers. Biotech animals (including insects) have thus far had very limited impact globally. To meet the international challenges of satisfying the increased demand for animal products sustainably in a warming world with shrinking resources, farmers need to have access to new technologies on a scale not yet remotely achieved.

Today we find ourselves at a crossroads for global policies regarding GnEd of agricultural products. Global dialog and collaboration are needed as we seek to create international trade and regulatory environments that facilitate the use of innovative precision breeding technologies, such as GnEd, within the animal sectors. Countries will need to implement change if we are to step forward with new hope for the future of sustainable agriculture and to encourage innovation and to ensure that solutions that have been developed are available to farmers within the needed timeframe to address the problems for which they were developed.

To do this, scientists, the private sector, and regulators, we need to work together and engage in further dialogue–because the next-generation will need more options, not fewer, to meet our growing agricultural needs and challenges and achieve the goal of a more sustainable and resilient animal agriculture.
